# The Impact of Financial and Psychological Wellbeing on Children’s Physical Activity and Screen-Based Activities during the COVID-19 Pandemic

**DOI:** 10.3390/ijerph18168694

**Published:** 2021-08-17

**Authors:** Louise C. Mâsse, Iyoma Y. Edache, Mark Pitblado, Sarah M. Hutchison

**Affiliations:** 1 School of Population and Public Health, University of British Columbia, Vancouver, BC V6T 1Z3, Canada; iyoma.edache@bcchr.ca (I.Y.E.); mark.pitblado@bcchr.ca (M.P.); 2 BC Children’s Hospital Research Institute, Vancouver, BC V5Z 4H4, Canada; shutchison@bcchr.ca; 3 Department of Pediatrics, University of British Columbia, Vancouver, BC V6H 3V4, Canada

**Keywords:** physical activity, screen time, youth, adolescents, health behaviors, social determinants, psychological wellbeing, pandemic

## Abstract

The COVID-19 pandemic, and the public health measures to combat it, have strained the finances of many families. While parents transitioned to working from home, children transitioned to learning virtually, limiting their organized social and physical activities. Families also reduced the frequency and size of gatherings, impacting psychological wellbeing. This study sought to understand the influence of financial wellbeing on children’s physical activity and leisure screen-based activities via mothers’ and children’s psychological wellbeing. In May and June of 2020, 254 Grade 7 Canadian children and their mothers completed separate online surveys assessing family financial wellbeing, mothers’ and children’s psychological wellbeing, and children’s physical activity and leisure screen-based activities. Structural equation modelling was used to examine the indirect effects of mothers’ and children’s psychological wellbeing on the relationship between financial wellbeing and children’s physical activity and leisure screen-based activities. Final models were adjusted for potential confounders. Study results indicate a significant indirect association between financial wellbeing and children’s physical activity and leisure screen-based activities via mothers’ and children’s psychological wellbeing. These findings demonstrate that higher levels of financial wellbeing are associated with better mental and physical health benefits in children during the COVID-19 pandemic.

## 1. Introduction

The unprecedented measures to limit the spread of the COVID-19 virus have made it difficult for children and youth to be physically active, in part, due to school closures and limited access to recreational facilities (e.g., parks and playgrounds) and organized sports [1]. Before the pandemic, accelerometry data indicated that only 39% of Canadian children and youth aged 5 to 17 years met the recommended physical activity guidelines [2,3]. Similarly, only 38% of Canadian children and youth met screen time guidelines [2,3]. Since the onset of the pandemic, further declines in physical activity among children have been documented [1,4,5]. A survey completed by a nationally representative sample of Canadian children reported that only 18.2% of children aged 5 to 17 years met the physical activity guidelines in April 2020 [4]. The lack of in-person interaction has also pushed children towards using screens for entertainment and social interaction [4,6]. One study reported that children were spending upwards of 6.5 h per day on leisure screen-based activities [4]. In addition to physical activity and screen-based behaviors, the population health measures used to curtail the spread of COVID-19 have also impacted children’s psychological wellbeing [7].

Growing evidence suggests the pandemic impacts children’s psychological wellbeing and indicates higher levels of anxiety and depression, especially for females [5,7,8]. Of particular concern is the effect of sustained isolation and significant loss of connectedness on children’s psychological wellbeing [9,10]. As higher anxiety levels during the pandemic have been associated with less physical activity (i.e., moderate to vigorous exercise), inactivity may have also contributed to the reported decline in psychological wellbeing [5]. In addition, the resources parents were able to provide their children during the pandemic may have also influenced their children’s psychological wellbeing [1]. Specifically, tangible resources, such as having a stable income to meet family needs or having the emotional capacity to support their children’s engagement in healthy activities during the pandemic.

The extent to which parents could meet their child’s needs during the pandemic may be related to the financial stress families experienced. During the pandemic, many people felt that their financial wellbeing was negatively impacted. This is not surprising as 15% of adults between the ages of 20 to 64 lost their employment, with women being disproportionately affected [11,12]. Although the Canadian government responded with income subsidies to mitigate the economic consequences of the pandemic [13], it is possible that financial insecurity still threatened Canadian families’ psychological wellbeing during the pandemic [1,14,15]. The financial impact of the COVID-19 pandemic was ranked, above getting ill with COVID-19, as the greatest contributor to the decline in psychological wellbeing during the pandemic [14]. A national survey among Canadian adults indicated that 80% of adults said that the pandemic negatively impacted their psychological wellbeing [14]. A greater increase in mental stress was reported in those who identify as females and those with two or more children [14,15]. Therefore, to understand the impact of COVID-19 on children’s emotional and physical wellbeing, it is essential to account for the familial context [16].

In the early stages of the pandemic, this study examined how the familial context, mainly mothers’ financial wellbeing and mothers’ psychological wellbeing, was directly and indirectly related to children’s psychological wellbeing and their involvement in physical activity and leisure screen-based activities.

## 2. Methods

### 2.1. Design and Setting

This study used data collected as part of the HABITs (Health and Behaviors in Teens) longitudinal study when children were in Grade 7. The data were collected from May to June 2020. This study was approved by the University of British Columbia Research Ethics Board (H15-01876).

### 2.2. Participants

Children in Grade 7 and their parents were recruited from 29 schools in the greater metropolitan area of Vancouver (British Columbia, Canada) for the HABITs study. After receiving school district approval, select schools were contacted to determine their interests in being part of the HABITs study. Schools were initially selected to ensure a diverse representation of visible minorities, educational backgrounds, and household incomes using the British Columbia Ministry of Education enrolment database, supplemented with census data. In schools that agreed to participate, research staff made presentations in Grade 7 classrooms and distributed information about the HABITs study in Fall 2019. To participate, children had to bring the informational packet home and return the signed parental consent form and their signed assent form. To be eligible to participate in the HABITs study, both the child and one of their parents had to complete the questionnaires in English. The study was designed to administer the questionnaires in early Spring 2020. However, due to the COVID-19 pandemic, children in Grade 7 and their parents were emailed separate links to the questionnaires in May and June 2020. A copy of the consent/assent form was included at the beginning of the questionnaire. All participants received a nominal incentive for their participation. Of the 401 families who initially signed up to participate in the HABITs study, we had complete data from 341 children and one of their parents. As the majority (75%) of the children participated with their mothers, this paper analyzed the children who participated with their mothers. In total 266 children participated with their mothers, but 12 pairs were omitted from the analyses as they had missing data on the main outcomes of this study, resulting in an analytical sample of 254.

### 2.3. Measures

Demographic information was collected from mothers and their children. Mothers were asked to report their age, marital status, ethnicity, education, total household pre-tax annual income, and employment status of themselves and their partners. Children self-reported their age and sex.

Financial Well-being was assessed in mothers with four items adapted from the US Consumer Financial Protection Bureau (CFPB) Financial Wellbeing Scale, measured on a five-point Likert scale (strongly agree to strongly disagree) [17]. The Scale was developed to quantify the extent to which someone’s financial situation and capability provides them with security and freedom of choice [17]. Items assessed the following: feeling of being in control of own finances, being able to handle unexpected expenses, feeling confident in meeting financial goals, and feeling pessimistic about meeting financial goals. In our sample, the Cronbach alpha was 0.81, demonstrating high internal consistency.

Self-esteem was measured using three items adapted from the General Self-Concept subscale of the Self-Description Questionnaire [18]. The items were measured on a five-point Likert scale (strongly agree to strongly disagree) and were: “I like being the way I am”, “I have lots to proud of”, and “There are lots of things about me that are good”. Higher scores indicate high levels of self-esteem. In our sample, the Cronbach’s alpha for mothers and children was 0.71 and 0.87, respectively.

Optimism was assessed using three items adapted from the Resiliency Inventory, which evaluates a positive perspective and outlook of the world and the future [19]. The items were measured on a five-point Likert scale (strongly agree to strongly disagree) and assessed: having more good times than bad times, believing more good things will happen, and positive thinking at the beginning of the day. Higher scores indicate high levels of optimism. In our sample, Cronbach’s alpha for mothers and children was 0.81 and 0.83, respectively.

Depressive symptoms were assessed in mothers with four items adapted from the Seattle Personality Questionnaire [20,21]. The items were measured on a five-point Likert scale (strongly agree to strongly disagree) and assessed: feeling happy a lot of the time, rarely getting upset about things, feeling things are right most of the time, and having lots of energy to do things. Higher scores indicate high levels of depressive symptoms. In this sample of mothers, the Cronbach alpha was 0.71.

Anxiety symptoms were assessed in children with three items adapted from the Seattle Personality Questionnaire [20,21] supplemented with three COVID-19 specific items. The items were measured on a five-point Likert scale (strongly agree to strongly disagree) and assessed worries about: what other kids might say, whether other people might like them, being teased, getting COVID-19, falling behind at school, and their family getting COVID-19. Higher scores indicate high levels of anxiety symptoms. In this sample of Grade 7 children, the Cronbach alpha was 0.80.

Leisure screen-based activities were measured with two items adapted from the Sedentary Behavior Questionnaire, a scale developed to assess the amount of time spent doing nine sedentary behaviors during a typical weekday and weekend day [22]. Children were asked to record the amount of time they spent watching movies, TV shows, or sports on a TV, computer, tablet, or mobile device during a typical weekday and weekend day. Children also reported the amount of time spent using a computer, tablet, or mobile device outside of schoolwork (e.g., using the internet, playing games, or texting on their phone) during a typical weekday and weekend day. For both items, response options were none, 15 min or less, 30 min, 1 h, 2 h, 3 h, 4 h, 5 h, or 6 h or more. Total weekly estimates of leisure screen-based activities were calculated by multiplying the weekday hours by 5 and weekend hours by 2, summing both values and dividing by 7.

Moderate-Vigorous Physical activity (MVPA) was measured with one item [23] that asked the following: “In a typical week, on how many days are you physically active for a total of at least 60 min per day (do not include school PE or gym class, but add up all the time you spent in any kind of physical activity)?” Categorical response options ranged from 0 days to 7 days per week.

### 2.4. Statistical Analysis

Descriptive analyses were performed using Stata version 17, (StataCorp LLC, College Station, TX, USA) [24]. Structural Equation Modeling (SEM) analyses were conducted in M-plus version 8.4, (Muthén & Muthén, Los Angeles, CA, USA) [25] to examine direct and indirect associations between mothers’ financial wellbeing and children’s health behaviors (i.e., MVPA and leisure screen-based activities), using mothers’ and children’s psychological wellbeing latent variables as indirect pathways. Mothers’ psychological wellbeing latent variable included scores on the following three scales: self-esteem, optimism, and depressive symptoms. Children’s psychological wellbeing included the scores on the following three scales: self-esteem, optimism, and anxiety symptoms. Mothers’ age and children’s sex were used as covariates in these models. In the analyses, both mothers’ and children’s psychological wellbeing were included as potential pathways in which financial wellbeing could indirectly influence children’s health behaviors (i.e., MVPA and leisure screen-based activities). The SEM analyses were broken down into two initial models to help understand the indirect effects. Model 1 examined the association between mothers’ psychological wellbeing and children’s behaviors with a potential indirect effect via children’s psychological wellbeing. Model 2 examined the association between financial wellbeing and children’s psychological wellbeing via mothers’ psychological wellbeing. Finally, Model 3 tested the full model, which combined the indirect pathways tested in Models 1 and 2. Given that the data was not severely skewed, the Maximum Likelihood estimation procedure using bootstrap procedures with 1000 replicates served to estimate the standard error and generate 95% confidence intervals (CI). Bootstrapped 95% CIs not straddling zero were considered statistically significant.

## 3. Results

Descriptive statistics of the analytic sample are presented in Table 1. The average age of mothers was 45.5 years; the majority were married or living common-law (85.3%) and were educated beyond the secondary level (92.5%). The proportion of mothers identifying as an ethnicity other than white (63.4%) was greater than those who identified as white (37.5%). About one-third of mothers reported an annual total household income of $150,000 or greater. Our sample of children was almost evenly split in terms of sex, with a slightly higher proportion of female children participating (54%). Almost all children in the sample were 13 years old at the time of participation. On average, children reported spending 5.7 h engaged in leisure screen-based activities per day and accumulated at least 60 min of MVPA on four out of seven days during a typical week.

Model 1 assessed whether mothers’ psychological wellbeing had a direct effect on their children’s MVPA and leisure screen-based activities or an indirect effect via children’s psychological wellbeing. The findings showed that mothers’ psychological wellbeing had a significant indirect effect on their children’s MVPA and leisure screen-based activities through their children’s psychological wellbeing (Table 2). Mother’s psychological wellbeing did not have a direct effect on the health behaviors of children.

Model 2 assessed the relationship between mothers’ financial wellbeing and children’s psychological wellbeing. Specifically, it assessed whether mothers’ financial wellbeing had a direct effect on their children’s psychological wellbeing or an indirect effect on children’s psychological wellbeing via mothers’ psychological wellbeing. Results showed that mothers’ financial wellbeing had a significant indirect effect on children’s psychological wellbeing via mothers’ psychological wellbeing (Table 2). The direct effect of mothers’ financial wellbeing on their children’s psychological wellbeing was not significant.

**Figure 1:**
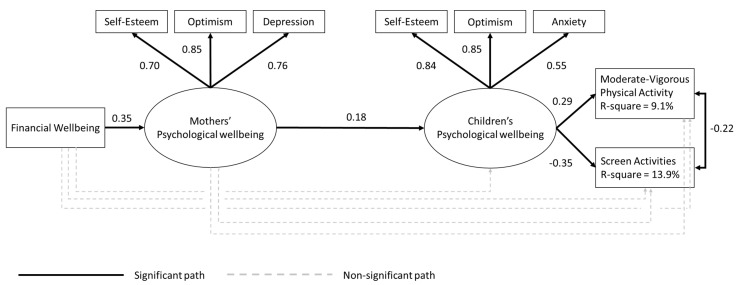
Financial wellbeing has an indirect effect on children’s health behaviors via mothers’ and children’s psychological wellbeing.

Model 3 assessed the three indirect pathways between mothers’ financial wellbeing and children’s health behaviors—specifically via the mothers’ psychological wellbeing, the child’s psychological wellbeing, or both. Results showed that mothers’ financial wellbeing had a significant indirect association with children’s MVPA and leisure screen-based activities through both mothers’ and children’s psychological wellbeing when considered together. However, mothers’ financial wellbeing did not have a direct effect on children’s health behaviors (Table 2). Figure 1 summarizes how financial wellbeing is indirectly associated with children’s MVPA and leisure screen-based activities via mothers’ and children’s psychological wellbeing together. Overall, the model in Figure 1 explained 9.1% and 13.9% of the variance of children’s MVPA and leisure screen-based activities, respectively.

## 4. Discussion

This study examined how the familial context was associated with children’s psychological wellbeing and their involvement in MVPA and leisure screen-based activities during the early stages of the COVID-19 pandemic (May–June 2020). The financial wellbeing of mothers indirectly affected their children’s MVPA and leisure screen-based activities via both mothers’ and children’s psychological wellbeing. Our findings uncovered a new pathway through which the COVID-19 pandemic negatively impacted children’s participation in physical activity and leisure screen-based activities. While it is recognized that the psychological wellbeing of families should be prioritized during and post-pandemic [26], this study underscores that financial wellbeing may be another way in which the COVID-19 pandemic had a negative impact on children’s MVPA and leisure screen-based activities, which must also be addressed to improve children’s health.

Our study found an association between financial wellbeing and mother’s psychological wellbeing. While previous pre-pandemic studies have also found similar relationships, reviews of longitudinal studies do not definitively confirm that financial insecurity or indebtedness contributes to the development of psychological wellbeing [27].This is likely because studies have assessed different aspects of psychological wellbeing (e.g., depression, anxiety) and inconsistently accounted for important confounders (e.g., baseline psychological wellbeing and reasons for financial stress) [27]. In contrast, studies that have examined job loss and psychological wellbeing found stronger associations with depression than anxiety, but employment benefits mitigated these relationships [28]. During the pandemic governmental subsidies were provided and aimed to mitigate any financial stress associated with the measures taken to curtail the pandemic, and to potentially reduce or eliminate the association with mothers’ psychological wellbeing. While we do not know whether this association existed before the pandemic, it is plausible to think that governmental subsidies may not have been enough to mitigate the relationship between financial wellbeing and psychological wellbeing, as the pandemic created a context of uncertainty which may have disproportionately impacted those with financial stress.

Previous studies have shown that mothers’ psychological wellbeing has deteriorated since the beginning of the pandemic [16,29] when there were huge social and economic disruptions in the home environment. In our sample, mothers’ psychological wellbeing was indirectly linked to children’s MVPA and leisure screen-based activities via children’s psychological wellbeing. Knowing that parents’ and children’s psychological wellbeing are linked, and they have a spillover effect on children’s health behavior (i.e., MVPA and leisure screen-based activities) stresses the need to consider the broader impact of quarantining and sheltering in place on children’s health. Evidence from previous infectious disease outbreaks suggests that financial stressors brought upon by the measures used to contain a pandemic may have a long-lasting impact on financial wellbeing of the family and psychological wellbeing of parents [26]. The pathway observed in this study, how financial wellbeing is associated with children’s MVPA and leisure screen-based activities via mothers’ and children’s psychological wellbeing, identifies another way in which the pandemic may indirectly impact children’s health.

One of the key findings of this study is the lack of a direct association between financial wellbeing and children’s participation in MVPA. This finding is in contrast to pre-pandemic studies that have found an association between family instrumental support (defined as the material ways in which parents support their children’s physical activity, such as the provision of equipment, enrollment in activities, and transportation to activities) and children’s participation in physical activity [30]. However, our study did not measure instrumental support but instead assessed financial wellbeing, which might explain why the results differed. Alternatively, the context during which data were collected for the present study might better explain why the direct association between financial wellbeing and children’s MVPA was not present. Precisely, data collection coincided with the beginning of the pandemic when children were out of school and businesses and workplaces were closed. At this specific time during the pandemic, the structured activities parents had relied upon to support their children’s involvement in physical activity vanished overnight (e.g., sports, extracurricular activities) and children’s movement behaviors were restricted [31]. As a result, participation in MVPA was no longer directly associated with having the financial resources to support children’s participation in physical activity as structured activities were upended. Based on these findings, post-pandemic families’ ability to support their children’s participation in physical activity might also depend on how quickly they can re-stabilize their financial situation and perhaps some families may require more support than others to ensure children remain physically active.

This study found a stronger association between children’s psychological wellbeing and leisure screen-based activities than physical activity behaviors. It is likely that during the pandemic, families became more reliant on screens to keep their children busy or to promote social interactions [6]. However, it remains that even in the early onset of the pandemic, when leisure screen-based activities likely provided some social interactions for children, high levels of screen-based activities were significantly associated with poor psychological wellbeing. Unsupervised leisure screen-based activities is particularly problematic during adolescence as it is a time when children are more susceptible to constant social comparisons, and interactions with social media may exacerbate the pressure to conform to social ideals [30]. Social media is thought to be one of the pathways that might explain the relationship between leisure screen-based activities and psychological wellbeing [32]. Our study findings highlight the importance of limiting screen-based activities to address children’s psychological wellbeing even during a pandemic.

The results of this study must be interpreted in light of its limitations. First, given that this is a cross-sectional study, causality cannot be ascertained. Second, the cross-sectional nature of the data also precluded a bidirectional investigation of how psychological wellbeing impacts health behaviors and, vice versa, how health behaviors impact psychological wellbeing. Third, children’s health behaviors were assessed via self-report as the research team was not permitted to send accelerometers to participants at this stage of the pandemic. The use of self-report to assess children’s health behaviors might have attenuated the relationships due to measurement errors. Fourth, while study participants were volunteers recruited in the greater metropolitan area of Vancouver, the generalizability of the findings might be limited to the context in which the pandemic evolved (e.g., subsidies were provided by the provincial government early in the pandemic). Importantly, participants were recruited and enrolled in the study well before any awareness of a pending pandemic (Fall 2019). As such, this study might have recruited a more representative sample of children in Grade 7. Finally, as our study sample consisted of only mothers, we do not know if these associations would be found in fathers.

## 5. Conclusions

In conclusion, the distinctive approaches used to curtail the impact of the COVID-19 pandemic were found to have a spillover effect on children’s physical activity and leisure screen-based activities among families that had financial stressors and poorer psychological wellbeing. In light of these findings, it will be essential to develop interventions that will support families that have been disproportionately affected by the pandemic. In addition, these interventions will likely need to take an integrative perspective that addresses the social determinants of health including financial wellbeing and gap in inequities to support children’s overall health and development.

## Figures and Tables

**Table 1:** Demographic characteristics of participants (*n* = 254).

Variable		% or Mean (SD)
**Mothers’ Characteristics**		
Mother’s age in years (*n* = 254)		45.5 (4.8)
Financial Wellbeing (*n* = 254)		3.7 (0.7)
Marital status (*n* = 251)	Not Married or common-law	14.7%
	Married or common-law	85.3%
Ethnicity (*n* = 254)	White	36.6%
	Chinese	24.8%
	Southeast Asian	4.7%
	Mixed	23.6%
	Other	10.3%
Education (*n* = 253)	High School or Less	7.5%
	Some College or Certificate	38.3%
	Bachelors, Graduate, or Professional Degree	54.2%
Total household income (*n* = 213)	Less than $50,000	21.6%
	$50,000–$99,999	22.1%
	$100,000–$149,999	23.9%
	$150,000 or higher	32.4%
Employment (*n* = 252)	Employed	71.4%
	Not Employed	28.6%
Employment of spouse (*n* = 211)	Employed	91.9%
	Not Currently Employed	8.1%
Mother psychological wellbeing (*n* = 254)	Self-esteem (1 = Low; 5 = High)	3.9 (0.6)
	Optimism (1 = Low; 5 = High)	4.0 (0.6)
	Depressive symptoms (1 = Low; 5 = High)	2.4 (0.6)
**Child Characteristics**		
Child sex: % female (*n* = 254)		54%
Child age in years (*n* = 254)		13 (0.1)
Leisure screen-based activities hrs/day (*n* = 245)		5.7 (3.7)
60 min of moderate-vigorous physical activity (days/week) (*n* = 254)		4 (2.3)
Child psychological wellbeing (*n* = 254)	Self-esteem (1 = Low; 5 = High)	4.0 (0.8)
	Optimism (1 = Low; 5 = Low)	3.7 (0.8)
	Anxiety symptoms (1 = Low; 5 = High)	3.0 (0.9)

Note: Percentages are calculated using the *n* associated with the variable. Participants were categorized as mixed ethnicity if they selected more than one of the ethnicity options. Total Household Annual Income is net income before taxes.

**Table 2:** Direct and indirect effects of financial wellbeing on children health behaviors via mothers’ and children’s psychological wellbeing.

Independent Variables	Mothers’ Psychological Wellbeing (PW)	Children’s PW	Physical Activity	Screen-Based Activities
	β (95% CI)	β (95% CI)	β (95% CI)	β (95% CI)
**Model 1 ^a^**	**Mediator**		**Dependent Variable**	
Children’s PW (Direct effect)			0.285 [0.138–0.424] *	−0.356 [−0.498–0.212] *
Mothers’ PW (Direct effect)		0.185 [0.048–0.317] *	−0.014 [−0.149–0.121]	0.027 [−0.130–0.175]
Mothers’ PW via Children’s MH (Indirect effect)			0.053 [0.013–0.114] *	−0.066 [−0.133–−0.019] *
**Model 2 ^b^**	**Mediator**		**Dependent Variable**	
Financial WB (Direct effect)	0.349 [0.195–0.480] *		0.021 [−0.116–0.165]	
Mothers’ PW (Direct effect)			0.178 [0.034–0.314] *	
Financial WB via Mothers’ PW (Indirect effect)			0.062 [0.015–0.134] *	
**Model 3 ^c^**	**Mediator**		**Dependent Variable**	
Financial WB (Direct effect)	0.349 [0.195–0.480] *	0.024 [−0.113–0.168]	−0.011 [−0.137 0.115]	−0.098 [−0.217–0.027]
Mothers’ PW (Direct effect)		0.178 [0.035–0.316] *	−0.011 [−0.155–0.140]	0.059 [−0.106–0.213]
Children’s PW (Direct effect)			0.286 [0.138–0.427] *	−0.354 [−0.495–−0.210] *
Financial WB via Mothers’ PW (Indirect effect)			−0.004 [−0.060–0.049]	0.021 [−0.037–0.083]
Financial WB via Children’s PW (Indirect effect)			0.007 [−0.032–0.055]	−0.008 [−0.062–0.040]
Financial WB via Mothers’ and Children’s PW (Indirect effect)			0.018 [0.004–0.045] *	−0.022 [−0.052–−0.005] *

^a^ Χ^2^ (df = 26) = 37.02, *p* = *0*.07; RMSEA = 0.041 (0.000–0.069); CFI = 0.98, SRMR = 0.039. ^b^ Χ^2^ (df = 21) = 31.59, *p* = 0.06; RMSEA = 0.045 (0.000–0.075); CFI = 0.98, SRMR = 0.036. ^c^ Χ^2^ (df = 29) = 38.58, *p* = 0.11; RMSEA = 0.036 (0.000–0.064); CFI = 0.98, SRMR = 0.033. * Bootstrapped 95% CIs not straddling zero which were considered statistically significant. β: Standardized estimate; CI: Χ^2^: Chi-square with *p*-value > 0.05 indicative of excellent and non-significant residuals; Confidence interval; RMSEA: Root Mean Square Error of Approximation, where upper confidence interval ≤0.08 is indicative of a good fit; CFI: Comparative Fit Index, where value >0.95 indicative of a good fit; SRMR: Standardized Root Mean Square Residual, where a value <0.05 indicative of a good fit. All models were adjusted for the following covariates: mother age and child sex. Child sex (female) was significantly associated with children’s psychological wellbeing (β = −0.244 CI = −0.364, −0.122).

## Data Availability

To access the data or any study materials please contact the corresponding author. The data are not made public as any data access has to be approved by the first author Research Ethics Board.
